# Impact of site of occlusion in proximal splenic artery embolisation for blunt splenic trauma

**DOI:** 10.1186/s42155-022-00315-0

**Published:** 2022-08-20

**Authors:** A. Boscà-Ramon, L. Ratnam, T. Cavenagh, J-Y Chun, R. Morgan, M. Gonsalves, R. Das, S. Ameli-Renani, V. Pavlidis, B. Hawthorn, N. Ntagiantas, L. Mailli

**Affiliations:** Department of Radiology, St. George’s Hospital, St George’s Healthcare NHS Trust, London, SW17 0QT UK

## Abstract

**Background:**

Proximal splenic artery embolisation (PSAE) can be performed in stable patients with Association for the Surgery of Trauma (AAST) grade III-V splenic injury. PSAE reduces splenic perfusion but maintains viability of the spleen and pancreas via the collateral circulation. The hypothesized ideal location is between the dorsal pancreatic artery (DPA) and great pancreatic artery (GPA). This study compares the outcomes resulting from PSAE embolisation in different locations along the splenic artery.

**Materials and methods:**

Retrospective review was performed of PSAE for blunt splenic trauma (2015–2020). Embolisation locations were divided into: Type I, proximal to DPA; Type II, DPA-GPA; Type III, distal to GPA. Fifty-eight patients underwent 59 PSAE: Type I (7); Type II (27); Type III (25). Data was collected on technical and clinical success, post-embolisation pancreatitis and splenic perfusion. Statistical significance was assessed using a chi-squared test.

**Results:**

Technical success was achieved in 100% of cases. Clinical success was 100% for Type I/II embolisation and 88% for Type III: one patient underwent reintervention and two had splenectomies for ongoing instability. Clinical success was significantly higher in Type II embolisation compared to Type III (*p* = 0.02). No episodes of pancreatitis occurred post-embolisation. Where post-procedural imaging was obtained, splenic perfusion remained 100% in Type I and II embolisation and 94% in Type III. Splenic perfusion was significantly higher in the theorized ideal Type II group compared to Type I and III combined (*p* = 0.01).

**Conclusion:**

The results support the proposed optimal embolisation location as being between the DPA and GPA.

## Background

In the setting of blunt splenic trauma, haemodynamically unstable patients are often managed surgically. Haemodynamically stable patients however undergo non-operative management (NOM) to preserve the spleen, its immune function and prevent overwhelming post-splenectomy infection (Ahuja et al. 2015; Harbrecht 2005; Peitzman et al. 2000). In these patients computed tomography (CT) is routinely performed, and injury severity is assessed by the American Association for the Surgery of Trauma (AAST) scale. Patients showing high AAST grade III-V splenic injury (Kozar et al. 2018), are frequently managed with adjunct splenic artery embolisation (SAE), which is associated with higher spleen salvage and lower NOM failure rates, especially in those with AAST grade IV-V splenic injuries (Quencer and Smith 2019; Banerjee et al. 2013; Crichton et al. 2017; Cinquantini et al. 2018; Bhullar et al. 2012).

SAE can be performed distally or proximally. Distal splenic artery embolisation (DSAE), consists of selective embolisation of branches distal to the hilum, and is usually carried out in patients with focal vascular injuries and has the additional benefit of preserving access for repeat intervention should this be required. However, DSAE is more technically challenging, takes longer and is associated with increased radiation dose and a higher rate of developing small splenic infarctions and life-threatening complications, such as rebleeding, abscess formation, and contrast-induced renal insufficiency (Patil et al. 2020; Clements et al. 2020; Imbrogno and Ray Jr 2012; Killeen et al. 2001; Schnüriger et al. 2011; Rong et al. 2017).

Proximal Splenic Embolisation (PSAE), involves embolisation of the main splenic artery (SPLA) and mimics surgical ligation. The premise is to reduce splenic perfusion pressure, while maintaining parenchyma viability via the development of collateral circulation, preserving spleen immune function, and preventing infarction or abscess formation (Bessoud et al. 2007; Anderson et al. 1977; Dc 1979; Zmora et al. 2009; Bessoud and Denys 2004). Over time, PSAE has become the preferred approach (Clements et al. 2020; Rong et al. 2017), especially in cases of multifocal injury or when no active bleeding is seen on angiography in splenic trauma.

**Fig. 1:**
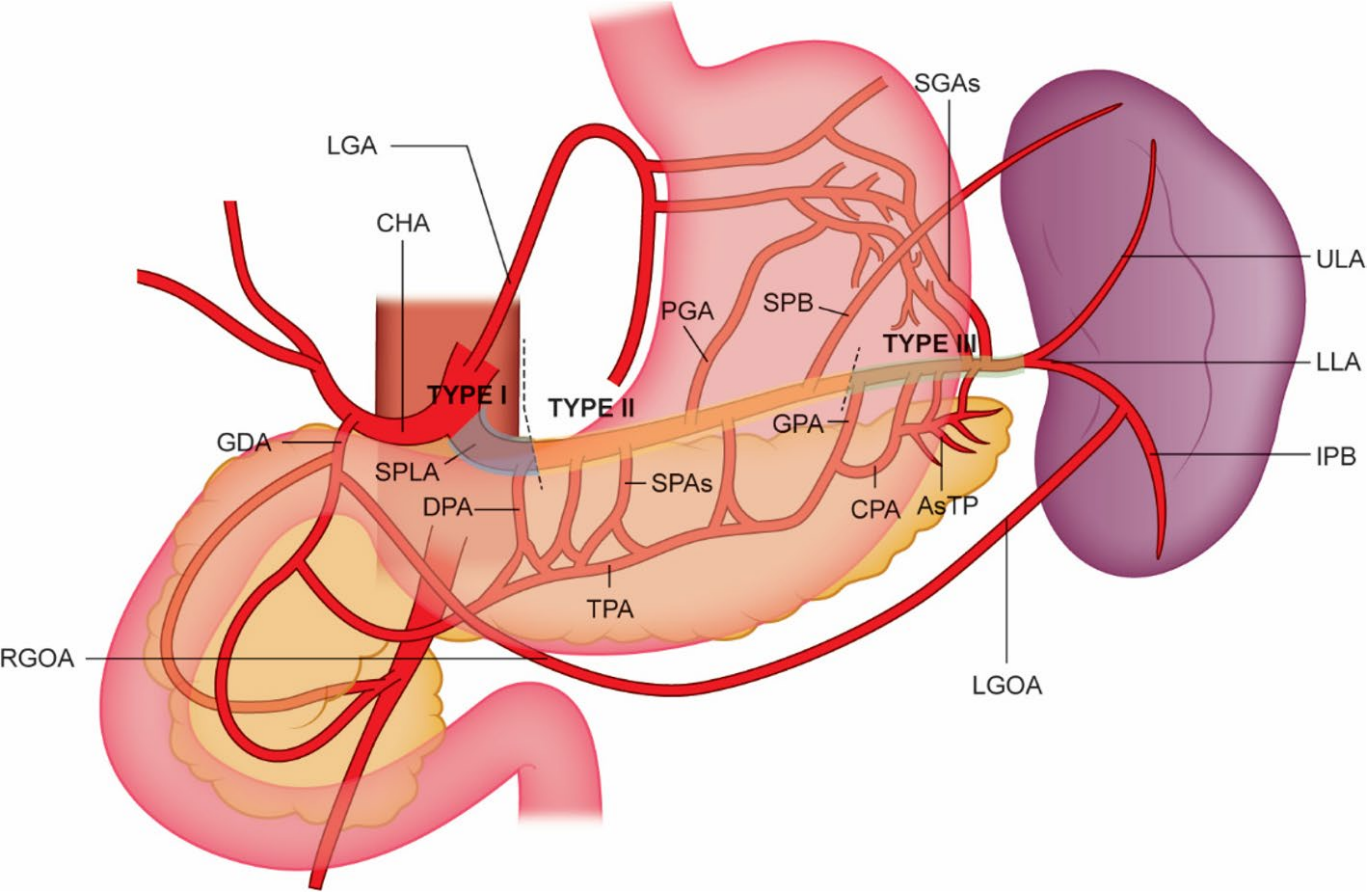
Arterial splenic supply. Arteries of the tail of the pancreas (AsTP), common hepatic artery (CHA), caudal pancreatic artery (CPA), great pancreatic artery (GPA), inferior polar branch (IPB), left gastric artery (LGA), left gastro-omental artery (LGOA), lower lobar artery (LLA), posterior gastric artery (PGA), right gastro-omental artery (RGOA), short gastric arteries (SGAs), short pancreatic arteries (SPAs), superior polar Branch (SPB), splenic artery (SPLA), transverse pancreatic artery (TPA), upper lobar artery (ULA)

Splenic artery branches supplying the pancreas from proximal to distal are the dorsal pancreatic artery (DPA), the short pancreatic arteries (SPAs), great pancreatic artery (GPA), and arteries of the tail of the pancreas (AsTP). Branches from the DPA, SPAs, and GPA anastomose to the transverse pancreatic artery (TPA) by forming three to four quadrangular arterial arcades. The AsTP however, anastomoses to the GPA via the caudal pancreatic artery (CPA). SPLA branches supplying the fundus and posterior regions of the stomach include the posterior gastric artery (PGA) and the short gastric arteries (SGAs), which establish anastomoses with branches of the left gastric artery (LGA), left gastro-omental artery (LGOA), and AsTP. The superior polar branch artery (SPB) usually originates from the distal SPLA and supplies the upper pole of the spleen. The SPLA at the splenic hilum then divides into the upper lobar (ULA) and lower lobar (LLA) arteries. Each lobar branch further divides into two to four lobular branches that enter the hilum of the spleen, which anastomose with one another (Daisy Sahni et al. 2003). A detailed outline of the arterial anatomy and supply is provided in Fig. 1.

The hypothesized ideal location for PSAE is between the DPA and GPA, in order to avoid devascularization of the pancreas and ischemic pancreatitis; as well as to preserve blood supply to the spleen (Ahuja et al. 2015; Quencer and Smith 2019; Patil et al. 2020; Imbrogno and Ray Jr 2012; Sclafani et al. 1991; Widlus et al. 2008; Ng et al. 2012). Although the use of PSAE instead of DSAE is now common practice, there is no evidence for the optimal embolisation location or outcomes from embolic material deployment along the different splenic artery segments. This study retrospectively evaluates a 5-year experience in PSAE following blunt splenic trauma in a major trauma centre. The aim of this paper is to evaluate the impact of site of occlusion in PSAE on technical and clinical outcomes, as well as on residual splenic perfusion and complication rates.

## Materials and methods

### Patient selection

The study period was over 5-years from 1st January 2015 to 1st January 2021. All patients above the age of 16 undergoing SAE for blunt splenic trauma were identified through the radiology information system (RIS) and included into the study. Patients presenting with penetrating splenic injuries, undergoing isolated diagnostic splenic angiograms, or isolated DSAE, or PSAE for other reasons than blunt splenic trauma, were excluded. All patients included in the study underwent an initial pre-procedural contrast enhanced CT.

### Data collection and definitions

Data was collected from RIS, picture archiving and communication system (PACS), and the electronic medical records (EMR). Information included demographics (age, gender), reported method of injury, initial CT information (date, time, findings, AAST splenic injury grade, solitary/multiple abdominal injuries), procedure information (date, time, type of embolic material utilized and location, complications, technical success), post-procedure progress (clinical success), complications (date, details), follow-up imaging (study type, date, findings, splenic perfusion).

Technical success was defined as significant blood flow reduction within the splenic artery at completion of the PSAE procedure. The demonstration of complete cessation of blood flow was not required. The development of collateral circulation was also utilised as a marker of significant blood flow reduction. Clinical success was defined as cessation of bleeding and spleen salvage after PSAE. This was deemed to be the case if the patient did not require reintervention and left the hospital with their spleen in situ or died from unrelated causes.

### Definitions of splenic artery anatomy and embolisation types

We designed a classification system where the main splenic artery was divided into three segments based on the DPA and GPA origins. After review of images on PACS, patients were assigned to one of the three categories based on the location of their embolisation:

- Type I: when performed proximally to DPA or covering its origin.
- Type II: when performed between DPA and GPA, not covering their origins.
- Type III: when performed distally to GPA or covering its origin.

**Fig. 2:**
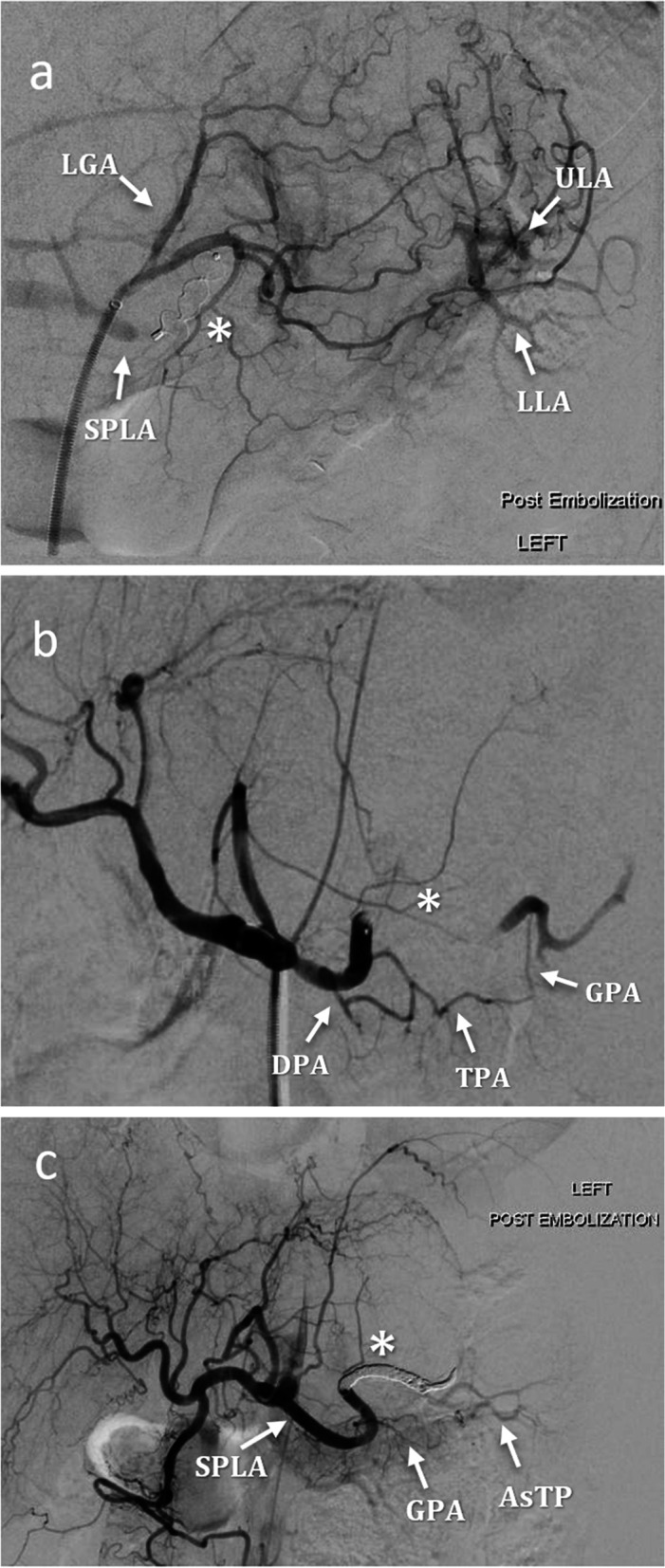
Types of PSAE. **a** Type I, AVP (white star) was deployed proximally to the DPA, note perfusion of the spleen via prominent LGA collateral circulation. **b** Type II, AVP (white star) inserted between DPA and GPA origin, spleen perfusion via collateral pancreatic circulation. **c** Type III, coiling (white star) distal to GPA, minimal splenic perfusion via AsTP

**Fig. 3:**
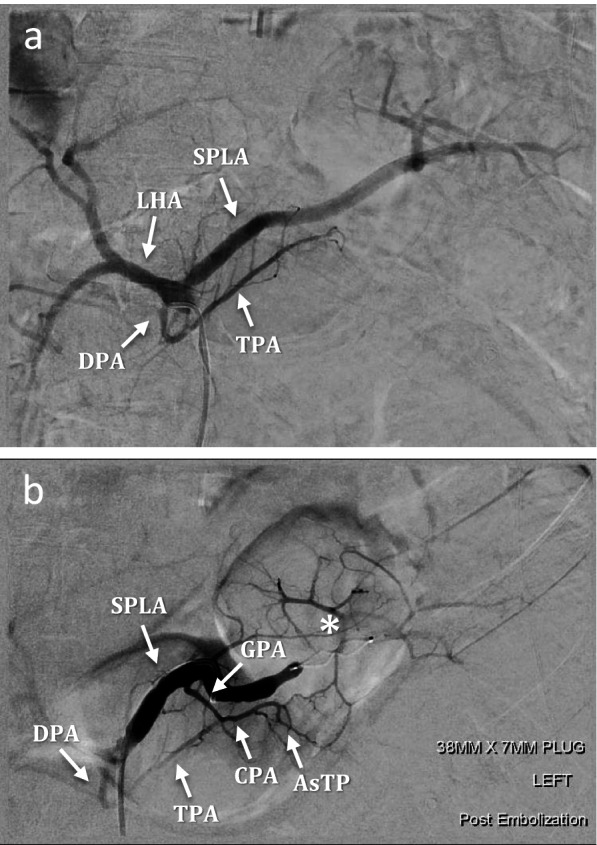
PSAE in patient presenting with blunt splenic trauma and variant splenic anatomy. **a** Pre-embolisation diagnostic angiogram revealed the DPA arising directly from the coeliac trunk along with the left hepatic artery (LHA). CT angiography showed the right hepatic artery origin from the superior mesenteric artery, and the left gastric artery arising from the coeliac trunk, not shown here. **b** Post-embolisation angiogram shows an AVP plug (white star) inserted distal to the GPA origin and splenic perfusion maintained via the AsTP collateral circulation. This case was thus considered a Type III embolisation

In this classification, Type II represents the ideal theorized location for embolisation, while Types I and III were the theorized locations to avoid (Fig. 2). When an anatomical variant was present, the same rules were applied. For example, if the DPA arose from the superior mesenteric artery, a Type I embolisation would not be possible as there would be no Type I location within the main splenic artery (Fig. 3).

### PSAE procedural technique

All procedures were performed by one of eight consultant interventional radiologists along with one of five interventional radiology fellows. The access was from the femoral artery, using either a 6-Fr 11 cm Radifocus™ introducer sheath (Terumo, Tokyo, Japan) or 6-Fr 35 cm Pinnacle® destination sheath (Terumo, Tokyo, Japan), a guiding 4-Fr or 5-Fr catheter (Cook Medical, Bloomington, USA) to selectively catheterize the celiac trunk and/or splenic artery, and when needed 2-Fr, 2.4-Fr, 2.7-Fr Progreat® microcatheters (Terumo, Tokyo, Japan). Embolic materials according to operator preference included 5-10 mm Amplatzer vascular plugs 4 (AVP 4) (St Jude Medical, Plymouth, USA), 5-14 mm pushable coils and detachable microcoils Concerto Helix (0.018″ Medtronic, Minnesota, USA), Ruby (0.018″ Penumbra, California, USA), POD (0.025″ Penumbra, California, USA), VortX (0.018″ or 0.035″ Boston scientific, Massachusetts, USA), and Gelfoam (Pfizer, Michigan, USA).

### Review of outcomes

All procedural angiograms were retrospectively reviewed and assessed for arterial anatomy and location of embolic agents. Both pre and post embolisation angiograms were reviewed. Patients who were imaged post embolisation had these assessed for evidence of pancreatitis and splenic perfusion. Post procedural blood parameters and clinical notes were reviewed for evidence of pancreatitis. Splenic perfusion was defined as the presence of contrast enhancement of the spleen on CT angiogram or the presence of a measurable Doppler signal on ultrasound, identified in > 50% of the splenic parenchyma.

### Statistical analysis

Statistical analysis was conducted using the Stata16 program. Two-sided *p*-value < 0.05 was chosen to indicate statistical significance, which was ascertained between groups using a chi-squared test.

## Results

A summary of patient and treatment demographics are shown in Table 1. Fifty-eight patients in total (43 males and 15 females; 18–95 years old, mean 51 years), underwent 59 PSAE. The number of patients assigned to each Type subgroup is as follows: 7 to Type I, 27 to Type II, and 25 to Type III. The median AAST splenic injury grade of all patients undergoing PSAE was IV. No patients with AAST grade I or II injuries underwent PSAE. The median time to embolisation was 0 days.

**Table 1:** Population and treatment demographics

Number of Patients	58
Number of embolisations	59
Age (mean, SD)	51 (21.76)
Gender (M:F, male percentage)	43:15 (76%)
AAST (median, range)	IV (III-V)
Time in days to embolisation (median, range)	0 (0–5)
Time in days to splenectomy after trauma (median, range)	7.5 (5–10)
Follow-up imaging (CT; US)	28; 6
Time in days to follow-up imaging (median, range)	3.5 (0–204)
Post embolisation preserved splenic perfusion (number, percentage)	33 (97%)

The most common cause of injury was road traffic accidents in 26 (45%), trauma secondary to a fall in 20 (34%), and workplace trauma in 5 (9%) patients. There were two post-surgical cases, one following a colonoscopy and the other post nephrectomy. Another patient presented with a bleed following cardiopulmonary resuscitation for an out of hospital cardiac arrest.

Full subgroup comparison data is shown in Table 2. Subgroups had comparable demographics. Median AAST grade was III for type I, IV for type II, and V for type III. PSAE in the ideal location between the DPA and GPA was performed in 46% of cases. Technical success was 100% in all subgroups. Clinical success was 100% for Type I and II embolisation and 88% for Type (22 out of 25 patients). Clinical success for Type II embolisation was significantly higher compared to Type III (*p* = 0.02). No significant difference was demonstrated on comparison between Type I with either Type II or Type III.

**Table 2:** Comparison of embolisation subgroups

	Number of patients	Number of embolisations	Age (mean, SD)	Gender (M:F, percentage)	AAST Grade			Time to embolisation (median, range)	Complications (number, percentage)	Splenectomy (number, percentage)	Follow-up imaging		Splenic perfusion (number, percentage)
					III	IV	V				CT	US	
Type I ≤DPA	7	7	47 (22.4)	6:1 (86%)	4	2	1	0 (0–1)	0 (0%)	0 (0%)	5	0	5 (100%)
Type II DPA-GPA	27	27	51 (22.9)	21:6 (78%)	8	12	7	0 (0–2)	0 (0%)	0 (0%)	7	4	11 (100%)
Type III ≥GPA	24	25	53 (20.9)	17:7 (71%)	4	8	13	0 (0–5)	3 (12.5%)	2 (8%)	16	2	17 (94%)

Use of embolic agents per subgroup is shown in Table 3. Coils as a single embolic agent were utilised in 29 patients, and vascular plugs in 25 patients. A combination of coils and vascular plug was used in 4 patients, and coils with gelatin sponge in only 1 patient.

**Table 3:** Embolic agent utilised per subgroup

	Coils	Vascular Plug	Coils + Vascular Plug	Coils + Gelatin Sponge
Type I ≤DPA	3	4	0	0
Type II DPA-GPA	7	19	1	0
Type III ≥GPA	19	2	3	1
Total	29	25	4	1

Follow-up imaging was carried out in 34 (59%) patients, the median time being 3.5 days, and in 33 (97%) of those patients, splenic perfusion was demonstrated. Splenic perfusion remained 100% in Types I (5 out of 5 patients) and II (11 out of 11 patients) embolisation and 94% in Type III (17 out of 18 patients). Splenic perfusion following Type II embolisation was significantly higher compared to Types I and III combined (*p* = 0.01). Needs to be noted, that the patient with a non-perfused spleen corresponded to a patient presenting with a shattered spleen (AAST grade V).

A total of 3 (5.1%) complications occurred, which consisted of two splenectomies and one reintervention for ongoing instability, all in the Type III embolisation subgroup. One of the patients initially underwent NOM, was discharged and readmitted 4 days after the initial trauma, presenting with increasing abdominal pain and ongoing instability. Repeat CT scan showed a large volume of haemoperitoneum and increased perisplenic haematoma. A decision was made to perform PSAE, however, the patient subsequently had a drop in his haemoglobin and required a splenectomy the following day. The other patient underwent PSAE on the same day of the trauma event but became unstable at day 8. Follow-up CT scan showed interval increase of perisplenic haematoma and a splenectomy was performed at day 10. The third patient presented with a re-bleed 5 days after Type III embolisation and underwent repeated more proximal Type III embolisation which was successful. The median time to splenectomy was 5 days.

## Discussion

Our series show an overall high technical (100%) and clinical (94.5%) success, as well as a low overall complications rate of 5.1%. Only 3.4% of patients requiring splenectomy. Clinical success was significantly higher for Type II embolisation compared to Type III. Splenic perfusion was significantly higher after Type II embolisation compared to Types I and III. Both splenectomy cases were in the Type III subgroup.

Our overall results are in line with previous studies of PSAE outcomes. A meta-analysis by Schnüriger et al. 2011 showed an overall rebleeding rate of 4.7–9% and splenectomy rate of 10.2%. The same study compared major complication rates between proximal and distal embolisations. Although there was a general tendency for higher complication rates with regards to re-bleeding, major infarction and infections following distal embolisation, the figures did not reach statistical significance. A more recent systematic review by Rong et al. 2017 reported a similar overall success rate of 90% and a major complication rate of 6.4%. The success rate was similar following proximal versus distal embolisation, however the incidence of major complications requiring surgical intervention was significantly higher following distal embolisation. Our results support this general consensus that proximal embolisation is more effective and safer than distal embolisation. There were no episodes of post-embolisation pancreatitis in our series, including patients in Type I embolisation. If the splenic artery is embolised proximal to the main pancreatic supply, usually the DPA, pancreatic ischemia may occur. It remains an unusual complication given the rich network of peri-pancreatic collaterals but a case report of severe acute necrotizing pancreatitis caused by inadvertent proximal embolisation reminds us to err on the side of caution (Hamers et al. 2009).

The demographics in our cohort of patients with a predominance of males (76%) and mean age of 51 years, are comparable to previous studies and correspond with road traffic accidents being the most common cause for these injuries (Waseem and Bjerke 2021; Pande et al. 2017). A case of active splenic bleeding after colonoscopy and one of splenic haematoma without parenchymal laceration after nephrectomy were also included in our study, as they were secondary to blunt injury mechanisms. Both are under recognized iatrogenic causes and mostly occur because of direct trauma, mobilisation, or traction of the spleen (Tan et al. 2011; Feola et al. 2016; Ullah et al. 2020; Skipworth et al. 2009).

In 2020 the number of PSAE decreased to 7 from a mean of 10 per year in previous years, moreover, none of them were related to road traffic accidents. This coincides with the start of the COVID-19 pandemic, when the first COVID-19 case in the UK was diagnosed on 31st January 2020 (Coronavirus: two cases confirmed in UK 2020), and lockdown restrictions were enforced from 26th March (Participation E 2021). During the COVID-19 pandemic, Kamine et al. 2020 reported a 57.4% decrease in overall trauma admissions and an 80.5% decrease in motor vehicle collisions, a trend also observed by Sutherland et al. 2020. We are uncertain if this had an impact on the outcome of embolisation as the mechanism of action for the splenic trauma differed from that most commonly seen.

Standard of practice at our centre as in many others, heavily favours PSAE over DSAE, mainly because PSAE is faster and less technically challenging (Imbrogno and Ray Jr 2012; Rong et al. 2017). This is particularly important in patients with unfavourable anatomy (difficult access, anatomical variation, and significant tortuosity), vasospasm, vascular disease, and the non-cooperative patient.

Vascular plugs were only used in 20% of Type III embolisations, compared to 57% in Type I and 70% in Type II. This is due to difficulties in deploying vascular plugs in the distal segment of the splenic artery due to the need to advance sheaths or larger catheters further into the arteries to enable their deployment. All other differences in embolic material reflect operator preference. The use of vascular plugs when compared to pushable coils, has shown similar high technical success and low complication rates. Previous studies prove that AVP 4 are suitable for achieving occlusion of the splenic artery, including redeployment as an advantage (Ng et al. 2012). Hypothetical complications such as delayed migration or partial recanalization (Pech et al. 2011; Zhu et al. 2011), did not occur in our cohort. Indeed, all three patients with complications in our series were embolised using coils alone. However, the choice of embolic agent was not felt to be the cause for the poor outcome in these cases. Rong et al. 2017, showed that the use of Gelfoam is associated with higher rates of severe complications and further surgical management compared to coils. Gelfoam use has also been associated with subsequent findings of intraparenchymal air on CT angiography (Ekeh et al. 2005; Haan et al. 2003).

In the 16 patients in which PSAE for AAST grade III splenic injuries was performed, no complications occurred, and none underwent splenectomy. Furthermore, 8 of these patients had follow-up imaging and in all of them splenic perfusion was preserved. This indicates that endovascular treatment of AAST grade III splenic injuries is safe with a low complication rate and adds evidence to the increasing trend to consider adjunct SAE to NOM in these patients.

As limitations, this is a retrospective study which inherently runs the risk of introducing bias. In some cases, the embolisation location was difficult to determine retrospectively on angiographic appearances. We acknowledge that tortuosity and variant anatomy is frequent within the splenic arterial territory which will influence choice and location of embolic material. Additionally, we note that the identification of the three splenic segments we propose in this paper is sometimes facilitated after embolic deployment and development of collateral circulation. Thus, identifying the ideal location may not be possible prior to embolisation. The assessment of splenic perfusion in this study was not uniform as routine post embolisation cross sectional imaging of the spleen with contrast was not carried out on all patients. We note that AAST grade distribution was different between groups, observing a higher median AAST grade of V in the type III embolisation group, which could have influenced the lower clinical success and perfusion rates. Preservation of immune function was also not assessed. The practices described in our paper are the reflection and preferences of a single major trauma centre. Despite the limitations of the study, these data represent real world outcomes confirming the safety of PSAE and further establishing its place in the NOM of splenic trauma. Errors of identification of the location of placement of embolic materials arising from the described limitations do not affect the overall clinical and technical success rates, however the outcome of this study has been to increase the awareness of the optimal location for placement of embolic materials and attempts to achieve this where feasible.

## Conclusion

The data presented supports the safety and efficacy of proximal splenic artery embolisation for blunt splenic trauma. The hypothesized ideal location for embolisation as between the DPA and GPA is shown to provide good clinical and technical success with significantly improved splenic perfusion compared to the other two locations before the DPA and after the GPA. PSAE for AAST III-IV grades after blunt splenic trauma is a safe adjunct option to NOM, in order to increase spleen salvage rates while preserving spleen viability. A good working knowledge of splenic arterial supply as illustrated in this paper will enable the interventional radiologist to place their embolic material where desired if technically feasible.

## Data Availability

The datasets used and/or analyzed during the current study are available from the corresponding author upon reasonable request.

## Abbreviations

AAST: American Association for the Surgery of Trauma
AsTP: Arteries of the Tail of the Pancreas
CHA: Common Hepatic Artery
CPA: Caudal Pancreatic Artery
CT: Computed Tomography
DPA: Dorsal Pancreatic Artery
DSAE: Distal Splenic Artery Embolisation
GPA: Great Pancreatic Artery
IPB: Inferior Polar Branch
LGA: Left Gastric Artery
LGOA: Left Gastro-Omental Artery
LHA: Left Hepatic Artery
LLA: Lower Lobar Artery
NOM: Non-Operative Management
PGA: Posterior Gastric Artery
PSAE: Proximal Splenic Artery Embolisation
RGOA: Right Gastro-Omental Artery
RIS: Radiology Information System
SAE: Splenic Artery Embolisation
SGAs: Short Gastric Arteries
SPAs: Short Pancreatic Arteries
SPB: Superior Polar Branch
SPLA: Splenic Artery
TPA: Transverse Pancreatic Artery
ULA: Upper Lobar Branch
US: Ultrasound
